# University College Hospital

**Published:** 1906-06-09

**Authors:** 


### UNIVERSITY COLLEGE HOSPITAL.

The North London or University College Hospital has issued the following appeal, which is signed by the Duke of Bedford, President of the hospital, Lord Monkswell, Treasurer, and Mr. Henry Lucas and Mr. Walter Baily, Chairman and Vice-Chairman of the hospital Committee: "It is with much regret that the Committee find themselves compelled to appeal again for funds to enable them to carry on the hospital. There is owing to tradesmen at the present time, a total debt of upwards of £8,000. Last year the Committee were obliged to sell out a considerable amount of stock in order to provide for the maintenance of the hospital. The Committee are most anxious to avoid a further sale whereby the income of the hospital must be again reduced and the available funded property be almost exhausted. But unless means are forthcoming for the discharge of the debt, a further sale of stock will be inevitable. The support from the public is quite inadequate to meet the increased requirements for maintenance of the enlarged hospital, the ordinary donations received up to the end of April this year only amounting to £824 13s. The total annual expenditure is upwards of £27,000, and the reliable income, including grants from the King Edward's Fund and the Hospital Saturday and Sunday Funds, does not exceed £9,000. If the deficiency cannot be met it will be necessary to close some of the beds. The Committee cannot contemplate this step without a feeling of dismay. They most earnestly appeal for immediate and increased support to avert so serious a proceeding, knowing as they do that decreased in-patient accommodation would prove a serious loss to the sick poor who are constantly pressing for admission to the wards. In 1905 the hospital treated 65,751 in- and out-patients, and there seems to be every prospect of an equal pressure upon the various departments for this year. The Committee cannot too often urge upon the benevolent the excellent method of improving the permanent income of the charity by endowing beds or cots, to be named in perpetuity as desired. Payments for the purpose may be made in one sum, by instalments, or by bequest. Contributions will be gratefully received and may be forwarded to the bankers, Messrs. Coutts and Company, 440 Strand, W.C., or to the Secretary, Mr. Newton H. Nixon, at the hospital."

